# Cultivation of Head and Neck Squamous Cell Carcinoma Cells with Wound Fluid Leads to Cisplatin Resistance via Epithelial-Mesenchymal Transition Induction

**DOI:** 10.3390/ijms22094474

**Published:** 2021-04-25

**Authors:** Till Jasper Meyer, Manuel Stöth, Helena Moratin, Pascal Ickrath, Marietta Herrmann, Norbert Kleinsasser, Rudolf Hagen, Stephan Hackenberg, Agmal Scherzad

**Affiliations:** 1 Department of Oto-Rhino-Laryngology, Plastic, Aesthetic and Reconstructive Head and Neck Surgery, University Hospital Würzburg, D-97080 Würzburg, Germany; stoeth_m@ukw.de (M.S.); moratin_h@ukw.de (H.M.); ickrath_p@ukw.de (P.I.); kleinsasse_n@ukw.de (N.K.); hagen_r@ukw.de (R.H.); hackenberg_s@ukw.de (S.H.); 2 IZKF Research Group Tissue Regeneration in Musculoskeletal Diseases, University Hospital Würzburg and Bernhard-Heine Centrum for Locomotion Research, University of Würzburg, D-97070 Würzburg, Germany; m-herrmann.klh@uni-wuerzburg.de

**Keywords:** cell proliferation, wound fluid, epithelial-mesenchymal transition, cisplatin resistance, Interleukin, head and neck squamous cell carcinoma

## Abstract

Locoregional recurrence is a major reason for therapy failure after surgical resection of head and neck squamous cell carcinoma (HNSCC). The physiological process of postoperative wound healing could potentially support the proliferation of remaining tumor cells. The aim of this study was to evaluate the influence of wound fluid (WF) on the cell cycle distribution and a potential induction of epithelial-mesenchymal transition (EMT). To verify this hypothesis, we incubated FaDu and HLaC78 cells with postoperative WF from patients after neck dissection. Cell viability in dependence of WF concentration and cisplatin was measured by flow cytometry. Cell cycle analysis was performed by flow cytometry and EMT-marker expression by rtPCR. WF showed high concentrations of interleukin (IL)-6, IL-8, IL-10, CCL2, MCP-1, EGF, angiogenin, and leptin. The cultivation of tumor cells with WF resulted in a significant increase in cell proliferation without affecting the cell cycle. In addition, there was a significant enhancement of the mesenchymal markers Snail 2 and vimentin, while the expression of the epithelial marker E-cadherin was significantly decreased. After cisplatin treatment, tumor cells incubated with WF showed a significantly higher resistance compared with the control group. The effect of cisplatin-resistance was dependent on the WF concentration. In summary, proinflammatory cytokines are predominantly found in WF. Furthermore, the results suggest that EMT can be induced by WF, which could be a possible mechanism for cisplatin resistance.

## 1. Introduction

Every year more than 800,000 people worldwide develop head and neck squamous cell carcinoma (HNSCC) and more than half of these patients die from this disease [1]. Risk factors include nicotine and alcohol consumption as well as infection with human papilloma virus [2]. Treatment strategies include primary surgery, radiotherapy, or the combination of these modalities.

Already during surgical tumor resection, the wound healing process starts. This is orchestrated by the activation of various immune cells, such as neutrophils, macrophages, and natural killer cells, but also of stromal cells, such as fibroblasts or mesenchymal stem cells and secretion of different cytokines and growth factors [3,4]. Many of these components are also found in surgical wound fluid (WF). Furthermore, cells in the area of the wound healing process undergo both functional and phenotypic changes [5]. One component of phenotypic change during wound healing is the epithelial-mesenchymal transition (EMT). During EMT, epithelial cells acquire mesenchymal properties due to biochemical changes. Here, the cells gain the ability to detach from their physiological cell complex, break through the basement membrane, and penetrate into the surrounding tissue as well as into lymphatic and blood vessels, where they adapt to new conditions [6,7]. EMT occurs in physiological processes such as embryogenesis or wound healing but also in pathological conditions such as tumor metastasis [8,9]. Various factors and signal pathways can induce an EMT. These include transforming the growth factor β (TGF-β), bone morphogenetic protein (BMP), epidermal growth factor (EGF), Wnt, Sonic Hedgehog (SHH), Notch, and integrin signaling [10,11,12]. EMT-inducing transcription factors that mediate this process include Twist1/2, SNAI1/2 (SNAI2 is also known as Slug), and zinc finger E-box-binding homeobox 1/2 (ZEB1/2) [13]. Since EMT is a distinct part of wound healing, it should be of interest with regard to cancer surgery. In most cases of HNSCC, only locoregional tumor growth is present at the time of primary diagnosis, and rarely distant metastasis may occur [14]. However, it should be noted that as soon as a distant metastasis is manifested, the prognosis is significantly attenuated [15]. In fact, more than 90% of cancer deaths are caused by tumor cells that have learned to leave their cell compound and spread throughout the body and establish distant metastases [16]. Thus, a key process in the cancer cells’ dissemination might be the EMT. Hence, EMT can promote tumor progression by increasing cell motility and invasion, which might support tumor dissemination in the body. Another important clinical aspect is the generation of therapy resistance in tumor cells. There are numerous data in the literature that suggest a link between EMT and development of drug resistance [17,18,19]. For example, in colorectal carcinoma it has been shown that increased expression of Twist1 in tumor cells leads to a worse survival rate in patients treated postoperatively with oxaliplatin and 5-fluorouracil [20]. Similar results were obtained with esophageal carcinomas. Esophageal tumors of patients treated preoperatively with chemotherapy showed increased expression of SNAI1 and ZEB1. This increased expression was associated with a poor prognosis [21].

Based on the fact that WF contains many substances that are released during wound healing, we wanted to evaluate a possible EMT induction of tumor cells after cultivation with WF. Furthermore, it was of interest to us whether chemoresistance could be induced by the WF as well. A more detailed understanding of the postoperative wound healing process could potentially be used to develop strategies to prevent both EMT induction and chemoresistance after tumor resection.

## 2. Results

### 2.1. Analysis of WF and FCS Cytokine Contents

**Figure 1:**
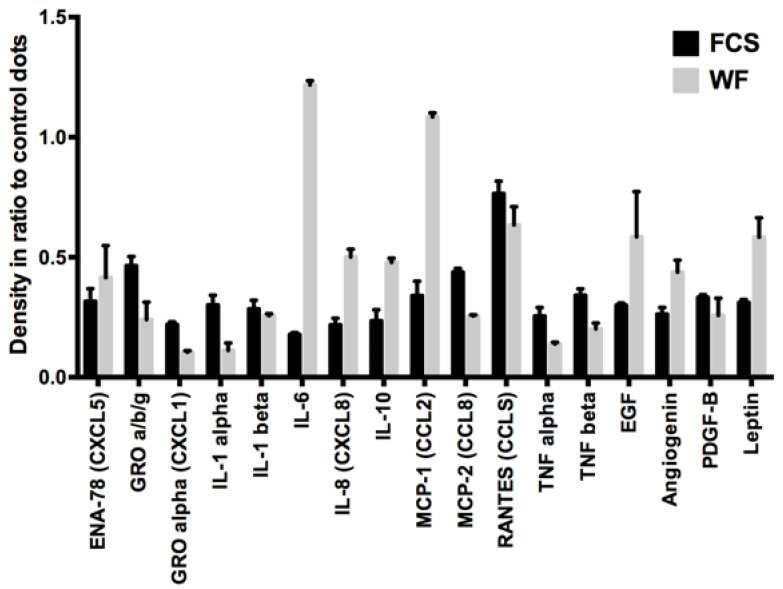
Semiquantitative cytokine contents analysis of pooled WF and FCS: In the WF compared with FCS, higher concentrations of IL-6, IL-8, IL-10, CCL2 (MCP-1), EGF, angiogenin, and leptin and lower concentrations of GRO, CXCL1, IL-1α, CCL8 (MCP-2), RANTES, TNFα, and TNFβ are detectable. Shown are the results of 10 pooled WF donors.

**Figure 2:**
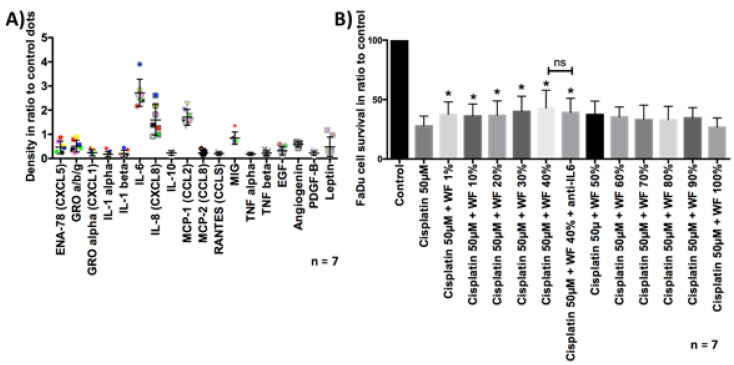
(**A**) Semiquantitative cytokine contents analysis of WF from all seven donors. One dot shows the result of one donor. High levels of IL-6, IL-8, MCR-1, MIG (monokine induced by gamma interferon; CXCL9) are detectable in WF compared with control dots. (**B**) FaDu cell survival was measured by flow cytometry. Previously, cells were treated with cisplatin 50 µM and ±WF at different concentrations. The cultivation with WF at a concentration of 1%, 10%, 20%, 30%, 40%, and 40% + anti-IL6 resulted in a significant improvement of cell survival after cisplatin treatment, compared with cultivation with expansion medium (*n* = 7). The addition of anti-IL6 had no statistically significant effect on cell survival. * *p* < 0.05. n.s. marks the not significant results.

First, the different cytokines in WF and fetal calf serum (FCS) were analyzed. For this purpose, a dot blot assay was performed. Cytokines responsible for angiogenesis, proliferation, inflammation, and immune modulation were examined. The results of the dot blot assays were quantified densitometrically and presented graphically (see Figure 1). We could show distinct differences between WF and FCS. Factors that were considerably more present in WF than in FCS were interleukin (IL)-6, IL-8, IL-10, chemokine (C-C motif) ligand 2 (CCL2) (monocyte chemoattractant 1 (MCP-1)), EGF, angiogenin, and leptin. Factors that were clearly less present in WF than in FCS were growth related oncogene (GRO), chemokine (C-X-C motif) ligand 1 (CXCL1), IL-1α, CCL8 (MCP-2), RANTES, tumor necrosis factor α (TNFα), and TNFβ. The cytokine concentrations differed between the donors. However, overall clustering of cytokine expression was comparable between the different individuals (see Figure 2).

### 2.2. Analysis of Cell Proliferation in Dependence of WF Concentration

**Figure 3:**
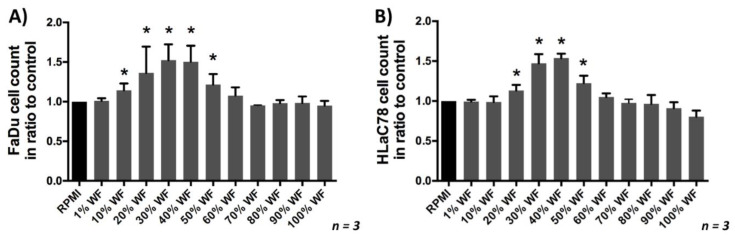
(**A**) FaDu and (**B**) HLaC78 growth in dependence of the WF concentration: The maximum cell proliferation in dependence of WF concentration was reached by incubating with RPMI medium supplemented with 30% and 40% WF (*n* = 3). Significant differences compared to the control (RPMI) were marked with an *.

To investigate the effect of WF on cell proliferation, a cell count was performed in dependence of WF concentration. We were able to show that with an increase in WF concentration, cell proliferation also significantly improved; the maximum proliferation performance was reached at about 30–40% WF. At higher concentration a decrease of proliferation occurred again, (see Figure 3). Further investigations were therefore carried out with 40% wound secretion.

### 2.3. Analysis of the Cell Cycle

**Figure 4:**
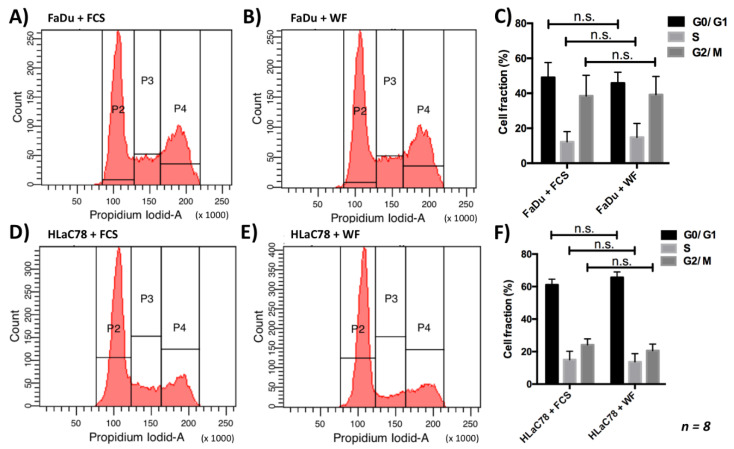
There is no change in the cell cycle distribution by incubation of (**A**–**C**) FaDu and (**D**–**F**) HLaC78 cells with 40% WF compared with incubation with FCS. This experiment was repeated with WF of 8 different donors. n.s. marks the not significant results.

Since the WF had an effect on cell proliferation, we carried out an analysis of the cell cycle. Here the different cell cycle phases (G0/G1, S, G2/M) after cultivation with WF and FCS were compared. The statistical analysis showed no significant difference between the different groups (see Figure 4) (*n* = 8).

### 2.4. Analysis of the Cell Morphology

**Figure 5:**
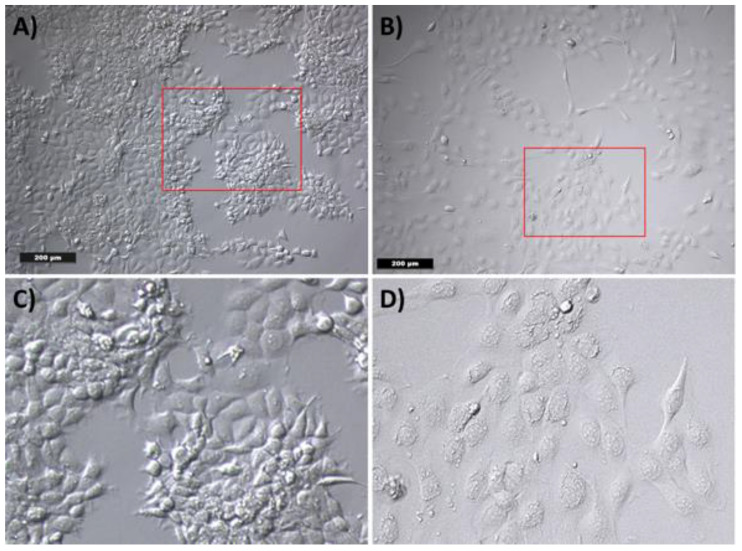
Cell morphology of tumor cells by incubation with (**A**) RPMI-EM and (**B**) WF. (**C**) and (**D**) show a digital magnification of the red marked area of (**A**) and (**B**), respectively. After incubation with (**A**) RPMI-EM, the tumor cells show a polygonal epithelial appearance. An elongated fibroblast-like cell morphology was observed after incubation with WF.

In order to evaluate the morphological effects of the medium on tumor cells, a cultivation of the tumor cells with WF for 24 h was performed. Evaluation of cell morphology was carried out with transmitted-light microscopy. The cultivation of the tumor cells with RPMI-expansion medium (RPMI-EM) served as a control. The tumor cells showed the typical polygonal epithelial structure. In contrast, the WF caused a change in the morphology of the tumor cells. Most of the tumor cells had a rather elongated fibroblast-like appearance (see Figure 5).

### 2.5. Analysis of EMT Markers after Incubation with WF

**Figure 6:**
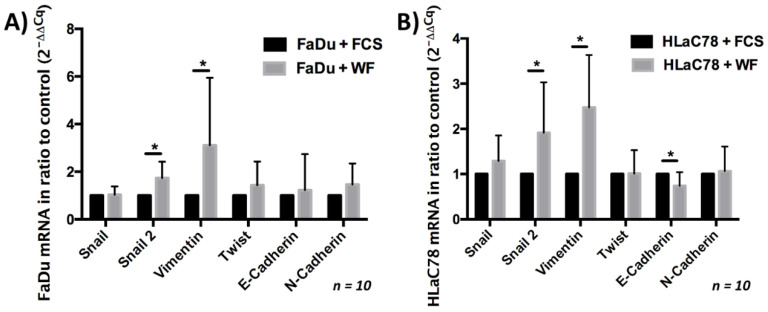
Expression of epithelial and mesenchymal markers after (**A**) FaDu and (**B**) HLaC78 incubation with WF. The expression of the mesenchymal markers Snail 2 and vimentin is significantly enlarged after incubation with WF in (**A**) FaDu and (**B**) HLaC78. Significant lower levels of the epithelial marker E-cadherin were detected in HLaC78 after incubation with WF (* *p* < 0.05).

The change in cell morphology suggested the evaluation of a possible EMT induction. This investigation was performed with rt-PCR. Both mesenchymal and epithelial markers were evaluated. We could show that WF significantly increased the expression of Snail 2 and vimentin in both tumor cell lines. In contrast, the expression of E-cadherin after cultivation with WF was reduced only in HLaC78 (see Figure 6).

### 2.6. Analysis of Chemoresistance

**Figure 7:**
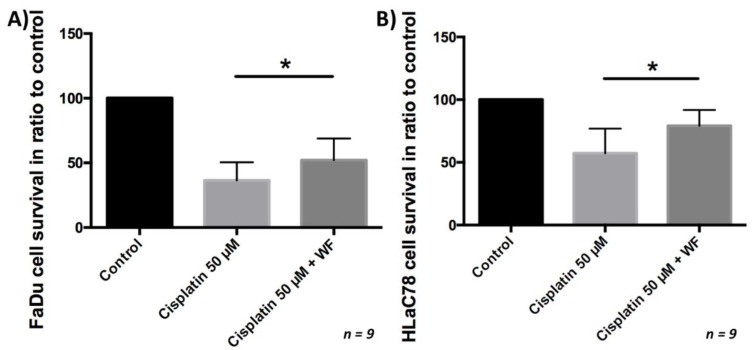
(**A**) FaDu and (**B**) HLaC78 cell survival measured by flow cytometry after incubation with cisplatin 50 µM and ±WF. There is significantly higher cell survival by cultivation of (**A**) FaDu and (**B**) HLaC78 with medium containing WF (*n* = 9).

After the tumor cells were cultivated with WF for 24 h, they were exposed to cisplatin for another period of 24 h. Subsequently, the vitality was analyzed by flow cytometry. We were able to show that significantly more cells were viable after cisplatin exposure and simultaneous cultivation with WF compared with control. This phenomenon was measurable for both the FaDu and HLaC78 cell lines (see Figure 7). Cell viability after exposure to cisplatin is dependent on WF concentration (see Figure 2). Significantly higher cell survival was observed after incubation with WF at concentrations of 1%, 10%, 20%, 30%, 40%, and 40% with anti-IL6. The addition of anti-IL6 did not significantly reduce cell survival (see Figure 2).

## 3. Discussion

After the surgical procedure, the wound healing process begins with the involvement of various cells that manage and coordinate the complex tasks in a highly dynamic manner. Within the framework of these processes, different cells dominate, above all the fibroblasts. Through the secretion of various factors in an autocrine or paracrine manner, the communication between the cells and their environment is enabled as well. Some of the secreted factors include EGF, platelet-derived growth factor (PDGF), IL-1, and TNFα [22]. IL-1 and TNFα in particular are able to activate the transcription factor activator protein 1 (AP-1) in mesenchymal cells via different signaling pathways, such as MAP kinases. The activation of AP-1 specific target genes leads to the secretion of growth factors, which in turn have mitogenic effects [23]. In addition to the proliferation-promoting factors, cell changes are also necessary to promote wound healing. Myofibroblasts are activated in response to tissue damage. Their primary function is to repair lost or damaged extracellular matrix. TGF-β remains as the main growth factor causing fibroblast differentiation into myofibroblasts [24]. However, other growth factors and cytokines can stimulate differentiation into myofibroblasts. These include PDGF, the connective tissue growth factor (CTGF), MCP-1, TNFα, IL-1β, and IL-6 [25,26]. Myofibroblasts play a crucial role not only in wound healing but also in the biology of solid tumors. Solid tumors are composed of malignant and non-malignant cells, the so-called tumor stroma. The myofibroblasts in this stroma are called tumor-associated fibroblasts. Myofibroblasts also secrete various factors in the tumor tissue, which on the one hand cause an immune modulation. On the other hand, they induce an improvement in angiogenesis, a remodeling of the extracellular matrix, by the secretion of various factors such as TGF-β or matrix metalloproteinases (MMPs), which in turn induce tumor proliferation. Furthermore, tumor-associated fibroblasts promote an induction of EMT [26]. Several cytokines and growth factors, which are responsible for both wound healing and tumor progression, were also found in the WF of our investigated patient population. However, the amount of IL-6 was remarkably high. The high concentration of IL-6 can be explained by the pro-inflammatory reactions during wound healing. IL-6 also plays an important role in cancer progression. In HNSCC, the relationship between IL-6 and oncological outcome has been investigated in recent decades. In a study conducted by Mojtahedi and colleagues, serum levels of IL-6 were elevated in untreated patients with HNSCC [27]. These results are supported by further studies. Allen and his team examined serum samples from patients with oropharyngeal carcinoma for the presence of various factors, including IL-6, during and after chemoradiation. They were able to demonstrate a correlation between increased serum IL-6 concentrations and reduced disease-specific survival [28]. Furthermore, IL-6 is able to act via several classical protein kinase cascades, such as mitogen-activated protein kinase (MAPK) and phosphatidylinositol-triphosphate kinase (PI-3-kinase) [29]. In addition, IL-6 is a potent activator of signal transducers and activators of the transcription (STAT) factors STAT1 and STAT3, which play an important role in the progression of neoplasia [30,31]. More interestingly, IL-6 also plays a role in the formation of cisplatin resistance. Duan and colleagues investigated the development of cisplatin resistance in non-small cell lung cancer (NSCLC). They found that IL-6 induces cisplatin resistance in tumor cells by upregulation of anti-apoptotic proteins B-cell lymphoma 2 (Bcl-2) and myeloid cell leukemia 1 (Mcl-1) and DNA repair-associated molecules ATM kinase, checkpoint kinase 1 (CHK1), tumor protein 73 (TP73), p53, and ERCC1 [32]. Additionally, in HNSCC, increased IL-6 expression is associated with a worse prognosis and acquired cisplatin resistance. Gao and colleagues were able to confirm this fact after evaluating tumor mRNA of 399 HNSCC patients [33]. These results suggest the potential possibility of combining cisplatin with IL-6 inhibitors. Looking at the analysis of cytokines and growth factors from our patients, we found significantly high values for IL-6. Tumor cells incubated with WF had a lower apoptosis after cisplatin treatment than the control group. The high IL-6 concentration could explain the reason for the significantly better survival of the tumor cells. However, the cisplatin resistance we observed in dependence of the WF concentration could not be reversed significantly by adding anti-IL-6. Therefore, the high IL-6 concentrations cannot be the only explanation for the WF-mediated cisplatin resistance. Chemoresistance is induced at different levels. For example, through the induction of EMT. Some studies on drug resistance development have already given evidence of EMT involvement. Işeri and colleagues investigated gene alterations in paclitaxel, docetaxel, and doxorubicin resistant MCF-7 cells. In this study it was found that EMT was responsible for the induction of multi-resistance of MCF-7 cells by increased expression of EMT-associated markers [34]. Haslehurst and colleagues used a whole human genome array to study cisplatin sensitive and resistant cell lines. They found that in cisplatin-resistant cells, markers such as Snail, Slug, Twist2, and ZEB2, were highly expressed, whereas markers such as E-cadherin were downregulated [18]. These changes suggested EMT induction. Inhibition of Snail and Slug not only altered the mesenchymal phenotype of the cells but also increased sensitivity to cisplatin. In the cell lines in our work, EMT-like changes were induced by the cultivation with WF. On the one hand, there was a change in morphology; on the other hand, markers such as Snail and vimentin were expressed significantly more. Still, not all markers were changed compared with the control. However, the PCR only gives a snapshot, so other EMT-associated markers could possibly have been produced in the course of the test. In our work, the cultivation of cells with WF had no influence on the cell cycle. Other studies could show that a cell cycle change may be associated with cytostatic resistance. Guo and colleagues were able to show that a cell cycle change minimizes the uptake of 5-fluorouracil and thus protects the cells from apoptosis [35]. Cell cycle changes were not seen in our work, so this is probably not a cause for the better survival of the cells after cisplatin therapy and simultaneous cultivation with WF.

In summary, it should be mentioned that the cultivation of tumor cells with WF had a proliferation-increasing effect. Furthermore, an EMT was induced. Under the cultivation of cells with WF, resistance to cisplatin was developed. The increased secretion of IL-6 and the induction of EMT could possibly be the cause of cisplatin resistance. Of course, further studies, especially with the targeted inhibition of EMT and IL-6, will be necessary in the future to better understand the mechanisms of WF and tumor cell interaction.

## 4. Materials and Methods

### 4.1. Collection of WF and Analysis of Cytokine Contents

The study was approved by the Ethics Committee of the Medical Faculty of the University of Würzburg (Reference Number: 116/17). In addition, written informed consent was obtained from all subjects included in the study. The WF collection was done according to our working group’s protocol, which was previously described [30,36]. WF of 10 patients (aged 51–88 years, 9 males, 1 female) were collected 48 h after surgery from the Redon drainages of tumor patients who had to undergo a planned neck dissection at the Department of Oto-Rhino-Laryngology, Plastic, Aesthetic and Reconstructive Head and Neck Surgery at the University Hospital Würzburg. At first, WF was centrifuged at 1300 rpm for 10 min at 4 °C. This allowed the cell debris to be removed. In addition, a second centrifugation step was performed in leucosep medium (Ficoll Paque Plus GE Healthcare, Freiburg, Germany) to eliminate immune cells. Finally, a further filtration was performed with a 0.45 μm syringe filter (Sarstedt, Nümbrecht, Germany). In order to avoid bacterial infection, 100 units/mL penicillin and 100 μg/mL streptomycin (1% penicillin/streptomycin) were added. The WF was stored at −80 °C until use [30,36].

In order to detect the different cytokines and growth factors, first the WF of all donors was pooled and analyzed by the dot blot assay. Secondly, the WF of the different donors were analyzed separately. All reagents and materials used, including the C Series Human Cytokine Antibody Array 3 kit (cat. no. AAH-CYT-3-4), were supplied by RayBiotech Inc. (Norcross, GA, USA). The membrane of this array is coated with antibodies. First, the WF was added on the membrane and incubated for 30 min at room temperature (RT). Several washing steps followed to remove unbound proteins. In a further step, 1 mL of a second antibody, which was a biotin-conjugated and horseradish peroxidase-conjugated streptavidin (1:1000), was added. After 2 h of incubation at RT, the detection of the proteins was finally performed by chemiluminescence. For this purpose, a detection buffer and exposure to an X-ray film were used. The quantification was performed densitometrically with the software ImageJ (version 1.8.0_112, Rasband, W.S., ImageJ, U. S. National Institutes of Health, Bethesda, MD, USA) in relation to the control dots.

### 4.2. Culture of Human Carcinoma Cell Lines FaDu and HLaC78

In the current study, the HNSCC cell lines FaDu and HLaC78 were used [37,38]. Cells were grown in RPMI-EM, consisting of RPMI-1640 medium (Biochrom Ltd., Cambridge, UK) supplemented with 10% FCS (Linaris Blue, Wertheim-Bettingen, Germany), 100 U/mL penicillin, 100 µg/mL streptomycin, 1% sodium pyruvate (100 mM; Biochrom, Ltd.), and 1% non-essential amino acids (100-fold concentration, Biochrom Ltd., Cambridge, UK). Cells were cultured in flasks at 37 °C with 5% CO_2_. The replacement of the medium was carried out every other day, and passaging was performed after reaching 70–80% confluence by trypsinization (0.25% trypsin; Gibco; Thermo Fisher Scientific, Inc., Waltham, MA, USA). Experiments were performed using cells in the exponential growth phase.

### 4.3. Effect of WF on Cancer Cell Proliferation

Cell proliferation analysis was performed after 24 h of incubation with the WF. First, the tumor cells with an initial cell count of 0.03 × 10^6^ were seeded in 12-well plates. The cells were cultivated for 24 h with the expansion medium. After 24 h the medium was changed, and the cells were rinsed with PBS. Subsequently, the cells were cultivated with WF with different concentrations (1, 10, 20, 30, 40, 50, 60, 70, 80, 90, and 100%). After another incubation period of 24 h, the MTT assay (Sigma-Aldrich; Merck KGaA, Darmstadt, Germany) was used to determine cell viability. After WF treatment, a washing step with PBS was performed. Subsequently, 100 µL MTT solution (1 mg/mL) was added for 5 h at 37 °C. After removal of the MTT solution, 100 µL isopropanol was added for another 1 h at 37 °C. The photometric evaluation for the measurement of color conversion was performed at 570 nm (Titertek Multiskan PLUS MK II; Thermo Labsystems, Helsinki, Finland).

### 4.4. Analysis of the Cell Cycle

The 0.1 × 10^6^ tumor cells were incubated in 12-well culture plates with RPMI-EM for 24 h. Subsequently, the cells were washed with PBS. This was followed by the cultivation with RPMI medium containing 30% WF. RPMI-EM served as control. After 24 h and 48 h, the cells were detached with trypsin and centrifuged at 4 °C and 500× *g* for 5 min. The supernatant was discarded, and the cell pellet was fixed in 70% ethanol. After another centrifugation step, the cell pellet was treated with 500 µL propidium iodide staining solution (BD Biosciences, Franklin Lakes, NJ, USA) at 4 °C for 15 min. Subsequently, the flow cytometric measurement (FACS Canto BD Biosciences) was performed.

### 4.5. Analysis of Cell Morphology and EMT Induction

After 0.3 × 10^6^ cells were seeded in 6-well plates, the cells were incubated with WF for 24 h; the cell morphology was analyzed by microscopic examination. In addition to the morphological analysis, the EMT was examined using rt-PCR. The following primers were selected (all primers were purchased from Thermo Fisher Scientific): Snail (Cat. No. Hs00195591_m1), Snail 2 (Cat. No. Hs00950344_m1), vimentin (Cat. No. Hs00958111_m1), Twist (Cat. No. Hs01675818_s1), E-cadherin (Cat. No. Hs01023894_m1), N-cadherin (Cat. No. Hs00983056_m1). Glyceraldehyde-3-phosphate dehydrogenase (GAPDH, Cat. No. Hs02758991_g1) was used as a housekeeping gene. The first step was total RNA extraction with RNeasy Kit (Qiagen, Venlo, The Netherlands) according to the manufacturer’s protocol. This was followed by reverse transcription of the RNA into cDNA (Super Script Reverse Transcriptase, Invitrogen). For the PCR we used SYBR Green Real-Time-PCR-Master-Mix (Thermo Fisher Scientific, Waltham, MA, USA). The PCR protocol was as follows: for 40 cycles, 50 °C for 2 min, 95 °C for 15 s, and 60 °C for 1 min; the first denaturation step was 10 min. The 2^−ΔΔCq^ method was used to quantify the proteins [39].

### 4.6. Analysis of Cisplatin Resistance

The most commonly used chemotherapeutic agent in the treatment of head and neck carcinomas is cisplatin. To evaluate a possible induction of chemoresistance by WF, HLaC78 and FaDu (0.1 × 10^6^ cells in a 12-well-plate) were cultivated in 40% WF for 24 h. Afterward, the cells were treated with 50 µM cisplatin for another 24 h. For FaDu it was repeated with the following WF concentrations: 1%, 10%, 20%, 30%, 40%, and 40% + adding anti-IL6 (500 ng/mL; R&D Systems, Wiesbaden, Germany) 50%, 60%, 70%, 80%, 90%, and 100%. Previously, half of the maximum inhibitor concentration (IC50) for cisplatin was determined in a preliminary test (data not shown). Apoptosis and necrosis after WF and cisplatin treatment were evaluated by flow cytometry using an Annexin V-propidium iodide kit (Becton-Dickinson Bioscience, Heidelberg, Germany). All steps were performed according to the manufacturer’s protocol. Afterward, WF and cisplatin exposure cells were detached by trypsinization, washed with PBS, and resuspended with binding buffer. For staining, Annexin V-APC and propidium iodide (PI) were added. Flow cytometry (FACScanto, Becton-Dickinson) was used for fluorescence detection.

### 4.7. Statistical Analysis

After all data were transferred to standard spreadsheets; the analysis was performed using GraphPad Prism 6.0 software (GraphPad Software, Inc., La Jolla, CA, USA). First, the analysis determined whether the distribution was Gaussian. The unpaired Student’s *t*-test was used in the case of Gaussian distribution; otherwise, the Kruskal–Wallis test was performed. A *p* < 0.05 was evaluated as statistically significant and marked with an asterisk. All data are presented ± standard deviation (SD).

## Data Availability

The data presented in this study are available on request from the corresponding author.

## Abbreviations

HNSCC	head and neck squamous cell carcinoma
WF	wound fluid
EMT	epithelial-mesenchymal transition
IL	interleukin
FCS	fetal calf serum
TGF-β	transforming growth factor β
BMP	bone morphogenetic protein
EGF	epidermal growth factor
SHH	Sonic Hedgehog
ZEB	zinc finger E-box-binding homeobox
CCL	chemokine (C-C motif) ligand
MCP	monocyte chemoattractant
GRO	growth related oncogene
CXCL	chemokine (C-X-C motif) ligand
TNF	tumor necrosis factor
RPMI-EM	RPMI-expansion medium
PDGF	platelet-derived growth factor
AP-1	activator protein 1
CTGF	connective tissue growth factor
MIG	monokine induced by gamma interferon
MMP	matrix metalloproteinases
MAPK	mitogen-activated protein kinase
PI-3-kinase	phosphatidylinositol-triphosphate kinase
STAT	signal transducer and activator of the transcription
Bcl-2	B-cell lymphoma 2
Mcl-1	myeloid cell leukemia 1
CHK1	checkpoint kinase 1
TP73	tumor protein 73
RT	room temperature

## References

[1] Bray F., Ferlay J., Soerjomataram I., Siegel R.L., Torre L.A., Jemal A. (2018). Global cancer statistics 2018: GLOBOCAN estimates of incidence and mortality worldwide for 36 cancers in 185 countries. CA Cancer J. Clin..

[2] Kreimer A.R., Clifford G.M., Boyle P., Franceschi S. (2005). Human Papillomavirus Types in Head and Neck Squamous Cell Carcinomas Worldwide: A Systematic Review. Cancer Epidemiol. Biomark. Prev..

[3] Wynn T.A., Vannella K.M. (2016). Macrophages in Tissue Repair, Regeneration, and Fibrosis. Immunity.

[4] Wynn T.A. (2008). Cellular and molecular mechanisms of fibrosis. J. Pathol..

[5] Rodrigues M., Kosaric N., Bonham C.A., Gurtner G.C. (2019). Wound Healing: A Cellular Perspective. Physiol. Rev..

[6] Alkatout I., Kabelitz D., Kalthoff H., Tiwari S. (2008). Prowling wolves in sheep’s clothing: The search for tumor stem cells. Biol. Chem..

[7] Thiery J.P., Acloque H., Huang R.Y., Nieto M.A. (2009). Epithelial-Mesenchymal Transitions in Development and Disease. Cell.

[8] Yang J., Weinberg R.A. (2008). Epithelial-mesenchymal transition: At the crossroads of development and tumor metastasis. Dev. Cell.

[9] Kim D.H., Xing T., Yang Z., Dudek R., Lu Q., Chen Y.-H. (2017). Epithelial Mesenchymal Transition in Embryonic Development, Tissue Repair and Cancer: A Comprehensive Overview. J. Clin. Med..

[10] McCormack N., O’Dea S. (2013). Regulation of epithelial to mesenchymal transition by bone morphogenetic proteins. Cell. Signal..

[11] Taipale J., Beachy P.A. (2001). The Hedgehog and Wnt signaling pathways in cancer. Nature.

[12] Gonzalez D.M., Medici D. (2014). Signaling mechanisms of the epithelial-mesenchymal transition. Sci. Signal..

[13] van Staalduinen J., Baker D., Ten Dijke P., Dam H. (2018). Epithelial-mesenchymal-transition-inducing transcription factors: New targets for tackling chemoresistance in cancer?. Oncogene.

[14] Takes R.P., Rinaldo A., Silver C.E., Haigentz Jr M., Woolgar J.A., Triantafyllou A., Mondin V., Paccagnella D., De Bree R., Shaha A.R. (2012). Distant metastases from head and neck squamous cell carcinoma. Part. I. Basic aspects. Oral Oncol..

[15] Noguti J., De Moura C.F.G., De Jesus G.P.P., Da Silva V.H.P., Hossaka T.A., Oshima C.T.F., Ribeiro D.A. (2012). Metastasis from oral cancer: An overview. Cancer Genom. Proteom..

[16] Meseure D., Alsibai K.D., Nicolas A. (2014). Pivotal Role of Pervasive Neoplastic and Stromal Cells Reprogramming in Circulating Tumor Cells Dissemination and Metastatic Colonization. Cancer Microenviron..

[17] Lu S., Yu L., Mu Y., Ma J., Tian J., Xu W., Wang H. (2014). Role and mechanism of Twist1 in modulating the chemosensitivity of FaDu cells. Mol. Med. Rep..

[18] Haslehurst A.M., Koti M., Dharsee M., Nuin P., Evans K., Geraci J., Childs T., Chen J., Li J., Weberpals J. (2012). EMT transcription factors snail and slug directly contribute to cisplatin resistance in ovarian cancer. BMC Cancer.

[19] Tsukasa K., Ding Q., Yoshimitsu M., Miyazaki Y., Matsubara S., Takao S. (2015). Slug contributes to gemcitabine resistance through epithelial-mesenchymal transition in CD133+ pancreatic cancer cells. Hum. Cell.

[20] Zhu D.-J., Chen X.-W., Zhang W.-J., Wang J.-Z., Ouyang M.-Z., Zhong Q., Liu C.-C. (2015). Twist1 is a potential prognostic marker for colorectal cancer and associated with chemoresistance. Am. J. Cancer Res..

[21] Hara J., Miyata H., Yamasaki M., Sugimura K., Takahashi T., Kurokawa Y., Nakajima K., Takiguchi S., Mori M., Doki Y. (2013). Mesenchymal phenotype after chemotherapy is associated with chemoresistance and poor clinical outcome in esophageal cancer. Oncol. Rep..

[22] Barrientos S., Stojadinovic O., Golinko M.S., Brem H., Tomic-Canic M. (2008). Perspective Article: Growth factors and cytokines in wound healing. Wound Repair Regen..

[23] Angel P., Szabowski A. (2002). Function of AP-1 target genes in mesenchymal-epithelial cross-talk in skin. Biochem. Pharmacol..

[24] Hinz B. (2016). Myofibroblasts. Exp. Eye Res..

[25] Kis K., Liu X., Hagood J.S. (2011). Myofibroblast differentiation and survival in fibrotic disease. Expert Rev. Mol. Med..

[26] Yazdani S., Bansal R., Prakash J. (2017). Drug targeting to myofibroblasts: Implications for fibrosis and cancer. Adv. Drug Deliv. Rev..

[27] Mojtahedi Z., Khademi B., Hashemi S.B., Abtahi S.M.B., Ghasemi M.A., Fattahi M.J., Ghaderi A. (2011). Serum interleukine-6 concentration, but not interleukine-18, is associated with head and neck squamous cell carcinoma progression. Pathol. Oncol. Res..

[28] Allen C., Duffy S., Teknos T., Islam M., Chen Z., Albert P.S., Wolf G., Van Waes C. (2007). Nuclear factor-kappaB-related serum factors as longitudinal biomarkers of response and survival in advanced oropharyngeal carcinoma. Clin. Cancer Res..

[29] Yang L., Wang L., Lin H.-K., Kan P.-Y., Xie S., Tsai M.-Y., Wang P.-H., Chen Y.-T., Chang C. (2003). Interleukin-6 differentially regulates androgen receptor transactivation via PI3K-Akt, STAT3, and MAPK, three distinct signal pathways in prostate cancer cells. Biochem. Biophys. Res. Commun..

[30] Scherzad A., Gehrke T., Meyer T., Ickrath P., Bregenzer M., Eiter R., Hagen R., Kleinsasser N., Hackenberg S. (2019). Wound fluid enhances cancer cell proliferation via activation of STAT3 signal pathway in vitro. Oncol. Rep..

[31] Leu C.-M., Wong F.-H., Chang C., Huang S.-F., Hu C.-P. (2003). Interleukin-6 acts as an antiapoptotic factor in human esophageal carcinoma cells through the activation of both STAT3 and mitogen-activated protein kinase pathways. Oncogene.

[32] Duan S., Tsai Y., Keng P., Chen Y., Lee S.O., Chen Y. (2015). IL-6 signaling contributes to cisplatin resistance in non-small cell lung cancer via the up-regulation of anti-apoptotic and dna repair associated molecules. Oncotarget.

[33] Gao J., Zhao S., Halstensen T.S. (2016). Increased interleukin-6 expression is associated with poor prognosis and acquired cisplatin resistance in head and neck squamous cell carcinoma. Oncol. Rep..

[34] Işeri Ö.D., Kars M.D., Arpaci F., Atalay C., Pak I., Gündüz U. (2011). Drug resistant MCF-7 cells exhibit epithelial-mesenchymal transition gene expression pattern. Biomed. Pharmacother..

[35] Guo X., Goessl E., Jin G., Collie-Duguid E.S.R., Cassidy J., Wang W., O’Brien V. (2008). Cell cycle perturbation and acquired 5-fluorouracil chemoresistance. Anticancer Res..

[36] Scherzed A., Hackenberg S., Froelich K., Radeloff A., Technau A., Kessler M., Hagen R., Rak K., Koehler C., Kleinsasser N. (2011). The effect of wound fluid on adipose-derived stem cells in vitro: A study in human cell materials. Tissue Eng. Part C Methods.

[37] Rangan S.R. (1972). A new human cell line (FaDu) from a hypopharyngeal carcinoma. Cancer.

[38] Zenner H.P., Lehner W., Herrmann I.F. (1979). Establishment of carcinoma cell lines from larynx and submandibular gland. Arch. Otorhinolaryngol..

[39] Livak K.J., Schmittgen T.D. (2001). Analysis of relative gene expression data using real-time quantitative PCR and the 2^−∆∆Ct^ Method. Methods.

